# Posttraumatic growth, posttraumatic stress disorder and resilience of motor vehicle accident survivors

**DOI:** 10.1186/1751-0759-4-7

**Published:** 2010-06-24

**Authors:** Daisuke Nishi, Yutaka Matsuoka, Yoshiharu Kim

**Affiliations:** 1 Department of Psychiatry, National Disaster Medical Center, Tokyo, Japan; 2 Clinical Research Institute, National Disaster Medical Center, Tokyo, Japan; 3 Department of Adult Mental Health, National Institute of Mental Health, National Center of Neurology and Psychiatry, Tokyo, Japan; 4 CREST, Japan Science and Technology Agency (JST), Saitama, Japan; 5 Department of Neuropsychiatry, Graduate School of Medical Science, Kyushu University, Japan

## Abstract

**Background:**

Although some previous studies have suggested that posttraumatic growth (PTG) is comprised of several factors with different properties, few have examined both the association between PTG and posttraumatic stress disorder (PTSD) and between PTG and resilience, focusing on each of the factors of PTG. This study aimed to examine the hypothesis that some factors of PTG, such as personal strength, relate to resilience, whereas other factors, such as appreciation of life, relate to PTSD symptoms among Japanese motor vehicle accident (MVA) survivors.

**Methods:**

This cross-sectional study was performed with 118 MVA survivors at 18 months post MVA. Data analyzed included self-reporting questionnaire scores on the Posttraumatic Growth Inventory (PTGI), the Impact of Event Scale- Revised (IES-R), and the Sense of Coherence (SOC) scale, which is one of the most widely used scales for measuring resilience. Correlations between scores on the PTGI and IES-R, the PTGI and SOC scale, and the IES-R and SOC scale were established by calculating Spearman's correlation coefficients.

**Results:**

PTGI was positively correlated with both SOC and PTSD symptoms, in spite of an inverse relationship between SOC and PTSD symptoms. Relating to others, new possibilities, and personal strength on the PTGI were correlated positively with SOC, and spiritual change and appreciation of life on the PTGI were positively correlated with PTSD symptoms.

**Conclusions:**

Some factors of PTG were positively correlated with resilience, which can be regarded as an outcome of coping success, whereas other factors of PTG were positively correlated with PTSD symptoms, which can be regarded as signifying coping effort in the face of enduring distress. These findings contribute to our understanding of the psychological change experienced by MVA survivors, and to raising clinicians' awareness of the possibility that PTG represents both coping effort coexisting with distress and outcome of coping success.

## Introduction

Posttraumatic growth (PTG) has attracted considerable attention over the past decade and is expected to add a new perspective to psychotherapy [1]. PTG is defined as the positive psychological change experienced as a result of the struggle with highly challenging life circumstances [2]. It is natural to assume that PTG, by definition, would show a negative correlation with posttraumatic stress disorder (PTSD), but Tedeschi and Calhoun hold that PTG is distinct from PTSD and does not show the same decrease in distress [2]. In fact, many previous studies have shown a positive correlation between PTG and PTSD [3-5], while others have reported a negative correlation between PTG and psychological distress or probable PTSD [6,7].

In regards to the relationship between PTG and resilience, where the latter has generally been defined as stress coping ability in the face of adversity, Janoff-Balman has proposed the model of psychological preparedness [8], in which it is posited that PTG would help survivors to be less traumatized by subsequent tragedies. The notion of psychological preparedness coincides with that of the stress inoculation model [9] or vaccination, in which exposure to moderate stress serves as a protection against subsequent stressors [8]. Following this model, PTG should show a positive correlation with resilience. However, the fact that PTG can correlate positively with both PTSD and resilience is very interesting, because a negative correlation between PTSD and resilience is obvious [10].

These features of PTG have been explained by, for example, differences in the use of instruments for measuring PTG [1] and possible curvilinear associations between PTG and PTSD [11], but another possible explanation is that PTG is composed of several factors with different properties. The Posttraumatic Growth Inventory (PTGI), which is one of the most widely used scales for measuring PTG, is comprised of five factors: relating to others, new possibilities, personal strength, spiritual change, and appreciation of life [12]. Some theorists have posited that different factors of PTG arise through different pathways and that, perhaps, different models should be developed for each factor. According to Janoff-Balman, personal strength and new possibilities can best be understood as strength through suffering, while appreciation of life, spiritual change, and relating to others can best be understood as existential reevaluation [8]. Also, some theorists have conceptualized PTG as a positive outcome of the struggle survivors face; others have conceptualized it as a coping strategy or a form of coping effort in the face of enduring distress [1]. It is possible, therefore, that some factors of PTG might frequently be related to coping success, which itself is linked with resilience and other factors related to coping effort, which links in turn to PTSD. If this is true, the unique association of PTG with both PTSD and resilience can be partly explained. Some previous studies concur with this hypothesis, showing that only personal strength and new possibility of PTG were strongly correlated with openness [12], and that only relating to others, spiritual change, and appreciation of life had a positive association with all subscales of PTSD [13].

It would seem important to elucidate the property of each factor of PTG since a better understanding of each factor could help clinicians recognize both the patient's coping effort coexisting with distress and the outcome of their coping success. However, few previous studies have examined both the association between PTG and PTSD and between PTG and resilience, focusing on each of the factors of PTG. The aim of this study was to examine the hypothesis that some factors of PTG, such as personal strength, relate to resilience, whereas other factors, such as appreciation of life, relate to PTSD symptoms among Japanese MVA survivors.

## Methods

### Participants and Procedure

The data used in this study were collected as a part of the Tachikawa Cohort of Motor Vehicle Accidents (TCOM) Study, a prospective longitudinal study investigating the incidence and prediction of psychiatric illness in a sample of patients injured in MVAs [14]. The TCOM study was approved by the Institutional Review Board and Ethics Committee of the National Disaster Medical Center (NDMC), Tokyo.

Individuals admitted to the acute critical care center of the NDMC between May 30, 2004 and January 8, 2008 were consecutively recruited. The inclusion criteria were (a) MVA-related severe physical injury causing a life-threatening or critical condition, (b) age between 18 and 69 years, and (c) native Japanese speaking ability. The exclusion criteria were as follows: (a) diffuse axonal injuries, brain contusion, and subdural and subarachnoidal bleeding detected by computed tomography and/or magnetic resonance imaging; (b) cognitive impairment, defined as a score of < 24 on the Mini Mental State Examination; (c) currently suffering from schizophrenia, bipolar disorder, drug (non-alcohol) dependence or abuse, or epilepsy prior to MVA; (d) marked serious symptoms such as suicidal ideation or self-harm behavior, or a severe physical condition preventing the patient from tolerating the interview; and (e) living or working at a location more than 40 km from NDMC.

This study was performed at 18 months post MVA (median = 561.5 days, range: 442-700 days post MVA). The participants were asked to visit the NDMC or to return a completed self-report questionnaire in a stamp-addressed envelope. All participants received a gift voucher for their participation (1,000 JPY [10 USD]). Of the 300 participants in the TCOM study, 118 (39.3%) participated in this study.

### Assessment

The assessment battery included the following: (a) general socio-demographics, and history of psychiatric illness; (b) injury severity score (ISS) [15]; (c) the Posttraumatic Growth Inventory (PTGI) [12,13]; (d) the Impact of Event Scale-Revised (IES-R) [16-18]; and (e) the Sense of Coherence (SOC) scale [19-21].

The PTGI, which assesses PTG [12], measures the degree of change experienced in the aftermath of a traumatic event. PTGI is comprised of five factors: relating to others, new possibilities, personal strength, spiritual change, and appreciation of life, and consists of 21 items. The degree of PTG for each item is rated on a 6-point scale (range, 0-105). Reliability and validity of the Japanese version of the PTGI have been verified [13].

The IES-R assesses posttraumatic stress symptoms [16,17] by examining the level of symptomatic responses to a specific traumatic stressor (MVA in our study) in the past one week. Three core symptoms of PTSD, namely, re-experience, avoidance, and hyperarousal, can be measured using the IES-R. It consists of 22 items, and the degree of distress for each item is rated on a 5-point scale (range, 0-88). Reliability and validity of the Japanese version of the IES-R have been verified [18].

Among the diverse concepts measuring resilience, the SOC scale was used in this study because it is frequently used as a measure of resilience and one of the most previously studied concepts [19,20]. SOC consists of three interrelated components, namely, comprehensibility, manageability, and meaningfulness [19]. People with strong SOC are less likely to assess a given situation as dangerous or uncontrollable and more likely to consider it as challenging, and thus maintain good health even under strenuous life events. A systematic review showed that SOC is strongly related to health, especially mental health, and quality of life [22,23]. In addition, a previous study showed that SOC is negatively correlated with PTSD [24]. The scale consists of 29 items, and responses are to be given using a 7-point scale (range, 29-203). Reliability and validity of the Japanese version of the SOC have been verified [21].

### Statistical Analyses

Demographic and accident-related characteristics were compared between the participants in this study and those who dropped out of the assessment by use of the χ^2 ^test and Mann-Whitney test for categorical and continuous variables, respectively. Interitem reliability between the items of each factor of the PTGI, IES-R and SOC were established by calculating Cronbach's alpha coefficients. The correlations between the scores on the PTGI and IES-R, the PTGI and SOC scale, and the IES-R and SOC scale were established by calculating Spearman's correlation coefficients because of the PTGI, IES-R, and SOC score distributions.

Any association with independent variables and covariates were quantified using 95% confidence intervals (95% CI). All statistical analyses used two-tailed tests. Statistical significance was established at *p *< .05. All data analyses were performed using SPSS statistical software version 14.0J for Windows (SPSS, Tokyo, Japan).

## Results

Patients who dropped out of the study were more likely to be younger (U = 8626.00, *p *< .01), and have less severe injury (U = 8995.50, *p *= .02). They did not differ significantly from those who participated in terms of history of psychiatric illness.

Of the 118 participants, 33 (28.0%) were women and median age was 37.0 years (range 18-69, mean = 39.7), and 11 (9.3%) reported a past history of psychiatric illness. Median ISS was 9.0 (range 1-48), and median score on the PTGI, IES-R and SOC scale were 42.0 (range 0-96), 10.0 (range 0-70), and 131.5 (range 34-203) respectively. The Cronbach's alpha coefficients for overall PTGI, relating to others, new possibilities, personal strength, spiritual change, and appreciation of life were .95, .88, .87, .83, .65, and .61, respectively. Those for overall IES-R, intrusion, avoidance, and hyperarousal were .96, .95, .86, and .89, respectively, and those for overall SOC, comprehensibility, manageability, and meaningfulness were .93, .84, .81, and .85. respectively.

The relationships between PTGI and IES-R scores, PTGI and SOC scores, and IES-R and SOC scores are shown in Tables 1, 2, and 3, respectively. Spiritual change and appreciation of life were correlated with all PTSD symptoms, especially avoidance. Personal strength and relating to others were correlated with all SOC subscales, and new possibility was also positively correlated with total SOC. IES-R was inversely correlated with SOC.

**Table 1:** Relationship between PTGI and IES-R at 18 months after MVA (N = 118)

	IES-R subscale			
PTGI subscale	intrusion	avoidance	hyperarousal	total
				(Mean = 14.8, SD = 16.4)
Relating to others	0.05	0.21*	0.10	0.14
New possibilities	0.05	0.23*	0.17	0.18
Personal strength	0.00	0.17	0.07	0.10
Spiritual Change	0.20*	0.31**	0.22*	0.27**
Appreciation of life	0.24*	0.41**	0.29**	0.35**
				
PTGI total	0.10	0.28**	0.17	0.21*
(Mean = 41.2, SD = 22.6)				

PTGI = Posttraumatic Growth Inventory; IES-R = Impact of Event Scale-Revised; MVA = motor vehicle accident; *, p < 0.05; **, p < 0.01.

**Table 2:** Relationship between PTGI and SOC at 18 months after MVA (N = 118)

	SOC subscale			
PTGI subscale	comprehensibility	manageability	meaningfulness	total
				(Mean = 131.5, SD = 26.5)
				
Relating to others	0.27**	0.27**	0.46**	0.38**
New possibilities	0.16	0.20*	0.45**	0.31**
Personal strength	0.31**	0.32**	0.43**	0.40**
Spiritual Change	0.02	0.10	0.35**	0.17
Appreciation of life	0.03	0.05	0.31**	0.14
				
PTGI total	0.21*	0.24*	0.47**	0.34**

PTGI = Posttraumatic Growth Inventory; SOC = Sense of Coherence scale; MVA = motor vehicle accident; *, p < 0.05; **, p < 0.01.

**Table 3:** Relationship between IES-R and SOC at 18 months after MVA (N = 118)

	IES-R subscale			
SOC subscale	intrusion	avoidance	hyperarousal	total
				
Comprehensibility	-0.47**	-0.36**	-0.43**	-0.45**
Manageability	-0.45**	-0.37**	-0.49**	-0.47**
Meaningfulness	-0.35**	-0.21*	-0.37**	-0.33**
				
SOC total	-0.48**	-0.34**	-0.49**	-0.47**

IES-R = Impact of Event Scale-Revised; SOC = Sense of Coherence scale; MVA = motor vehicle accident; **, p < 0.01.

In sum, PTG was positively correlated with both SOC and PTSD symptoms, in spite of the inverse relationship between SOC and PTSD symptoms. The factors personal strength, relating to others, and new possibilities were correlated positively with SOC, and spiritual change and appreciation of life were positively correlated with PTSD symptoms.

## Discussion

These results of this study were mostly consistent with our hypothesis that certain factors of PTG relate to resilience, while others relate to PTSD symptoms. They are also consistent with the findings of a previous study involving MVA survivors in which those with PTSD scored higher in their perceptions of appreciation of life and spiritual change than those without PTSD, and those without PTSD scored higher in their perception of personal strength than those with PTSD [25].

In the present study, appreciation of life and spiritual change were strongly correlated with avoidance as measured by the IES-R. Avoidant coping can interfere with more active coping [4]. In addition, spiritual change and appreciation of life may lead to being aware of mortality and fragility [1], which could also support the view that these two factors may be coping efforts in the face of enduring distress. Clinicians are generally encouraged to validate the perception of PTG in patients [1]; however, identifying and being more attentive to the patient's own meanings and interpretations may be more beneficial, especially when patients experience changes in appreciation of life and spirituality.

On the other hand, the perception of personal strength, relating to others, and new possibility could be seen as the outcome of coping success or adaptive coping based on the results of this study. Although Janoff-Balman emphasized the commonalities among relating to others, appreciation of life, and spiritual change in that all of three factors stem from a new-found appreciation [8], Hiraki pointed out the commonalities between relating to others and new possibility in that both of these factors could be stimulated by finding new interests or new paths through social support [26]. Tedeschi and Calhoun, who created the PTGI, suggested that PTG is distinct from resilience, arguing that PTG is transformative whereas resilience is not [2]. Moreover, they suggested that people who are resilient might be unlikely to experience PTG because the traumatic experience might not be challenging to them [27]. However, Lepore and Revenson [28] described PTG as one form of resilience, in that resilience may develop from transformations. The results of this study might lend support to the notion that some factors of PTG can, at the very least, be regarded as forms of resilience. Future studies with a prospective design should seek to examine whether certain factors of the PTGI predict adaptation or even enhance resilience while others predict more PTSD symptoms over and above the initial PTSD severity.

All of the factors of PTG were shown to be related to meaningfulness of SOC. Calhoun and Tedeschi [29,30] described that the experience of a major life crisis leads to the individual engaging in a ruminative process: Deliberate rumination is involved in the development of PTG, whereas intrusive rumination is characterized as negative and unwanted thoughts. A previous study involving Japanese students also showed that deliberate rumination leads to PTG [31]. Deliberate rumination could be the cognitive process whereby one finds meaning in the traumatic event, which might explain the positive relationship between meaningfulness of SOC and PTG.

The mean PTGI score of the participants in this study was 41.2, which is comparable to that of previous studies of MVA survivors [25] and Japanese students [13]. The mean IES-R score was 14.8, which is also comparable to that of previous studies of accident survivors [32,33]. In addition, the mean SOC score, which is thought to be stable over time, was 131.5, which is almost the same as that found in the Japanese general population [21]. Overall, therefore, our sample can be viewed as representative of the Japanese general population in terms of PTGI and SOC scores, and comparable to previous studies regarding MVA survivors in terms of PTGI and IES-R scores.

This study has several strengths such as consecutive sampling, but also has some limitations. First, the sample size was modest compared to previous studies regarding PTG. Second, the attrition rate was relatively high. Third, the cross-sectional design of this study cannot reveal any causality. Fourth, correlation coefficients can be affected by many causes, such as the number of individual factors of PTG. Moreover, recent criticism of the PTGI pointed out that it did not appear to measure actual pre- to posttrauma change [34]. Accordingly, although the PTGI has been widely used, its utility should be examined further.

## Conclusions

The results of this study suggest that, for MVA survivors, some factors of PTG, such as personal strength, relating to others, and new possibility, are positively correlated with resilience represented by SOC, whereas other factors. such as appreciation of life and spiritual change, are positively correlated with PTSD symptoms. These findings contribute to our understanding of the psychological change experienced by MVA survivors, and to raising clinicians' awareness of the possibility that PTG represents coping effort coexisting with distress and the outcome of coping success.

## Competing interests

This work was supported by grants (Research on Psychiatric and Neurological Disease and Mental Health: 16190501, 19230701 and 2030701) from the Japanese Ministry of Health, Labor and Welfare, and Japan Science and Technology Agency, CREST. Funding sources did not play any other role. The authors did not have any competing interests in this work.

## Authors' contributions

DN participated in the design of the study, performed statistical analysis, and drafted the manuscript. YM conceived of the study and helped draft the manuscript. YK conceived of the study and revised the manuscript critically for important intellectual content. All authors read and approved the final manuscript.
